# Assessing wind field characteristics along the airport runway glide slope: an explainable boosting machine-assisted wind tunnel study

**DOI:** 10.1038/s41598-023-36495-5

**Published:** 2023-07-06

**Authors:** Afaq Khattak, Pak-wai Chan, Feng Chen, Haorong Peng

**Affiliations:** 1 Key Laboratory of Infrastructure Durability and Operation Safety in Airfield of CAAC, College of Transportation Engineering, Tongji University, 4800 Cao’an Road, Jiading, Shanghai, 201804 China; 2 Hong Kong Observatory, 134A Nathan Road, Kowloon, Hong Kong China; 3 The Key Laboratory of Infrastructure Durability and Operation Safety in Airfield of CAAC, Tongji University, 4800 Cao’an Road, Jiading, Shanghai, 201804 China

**Keywords:** Natural hazards, Environmental impact, Engineering, Civil engineering

## Abstract

Aircraft landings are especially perilous when the wind is gusty near airport runways. For this reason, an aircraft may deviate from its glide slope, miss its approach, or even crash in the worst cases. In the study, we used the state-of-the-art glass-box model, the Explainable Boosting Machine (EBM), to estimate the variation in headwind speed and turbulence intensity along the airport runway glide slope and to interpret the various contributing factors. To begin, the wind field characteristics were examined by developing a scaled-down model of Hong Kong International Airport (HKIA) runway as well as and the surrounding buildings and complex terrain in the TJ-3 atmospheric boundary layer wind tunnel. The placement of probes along the glide slope of the model runway aided in the measurement of wind field characteristics at different locations in the presence and absence of surrounding buildings. Next, the experimental data was used to train the EBM model in conjunction with Bayesian optimization approach. The counterpart black box models (extreme gradient boosting, random forest, extra tree and adaptive boosting) as well as other glass box models (linear regression and decision tree) were compared with the outcomes of the EBM model. Based on the holdout testing data, the EBM model revealed superior performance for both variation in headwind speed and turbulence intensity in terms of mean absolute error, mean squared error, root mean squared error and R-square values. To further evaluate the impact of different factors on the wind field characteristics along the airport runway glide slope, the EBM model allows for a full interpretation of the contribution of individual and pairwise interactions of factors to the prediction results from both a global and a local perspective.

## Introduction

### Wind shear and turbulence: the invisible enemies

**Figure 1:**
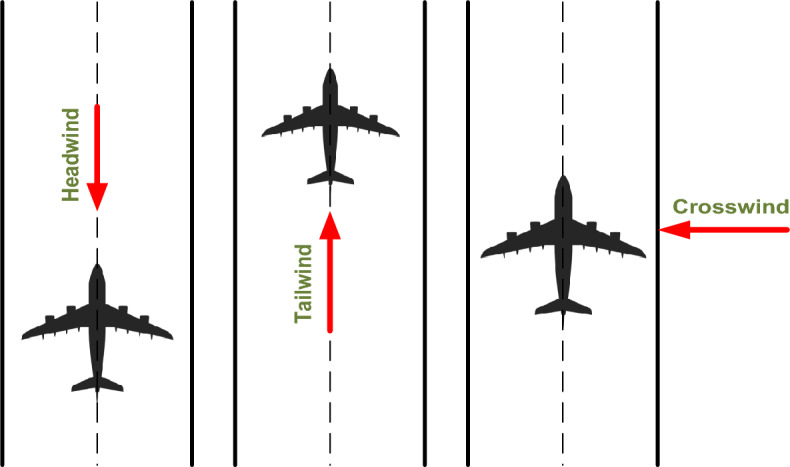
Type of wind effects on the departing and approaching aircraft.

The proximity of wind to an airport's runway has a significant impact on aircraft operations. It should come as no surprise that wind is one of the most important factors influencing the flight path of an aircraft. When discussing the "wind effect," it is important to understand the three broad classifications of wind types^1^. Wind can influence takeoff and landing from three different directions. When the wind is blowing in the direction of an aircraft, it is referred to as a “headwind”. When the wind is blowing from behind the aircraft, it is referred to as a “tailwind”. When the wind is blowing from the side of the aircraft, it is referred to as a “crosswind”. It is preferable for the aircraft to land in the direction of the wind, which facilitates the landing by reducing its speed. Likewise, it is preferable for the aircraft to take off in the wind's direction. During takeoff, a headwind increases airflow; consequently, the necessary lift is attained sooner and at lower speeds (the wind speed is added to the aircraft speed). Therefore, less runway is required for safe takeoff and landing. Likewise, the tailwind is preferred because it speeds up the aircraft and aids its propulsion systems, as shown in Fig. 1.

**Figure 2:**
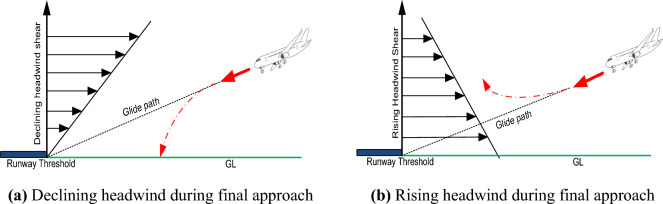
Detrimental Effects of wind shear on the approaching aircraft.

Constant wind speed is preferable from a takeoff and landing safety perspective. Consequently, wind speed is not a problem in and of itself. The issue arises, however, when the wind speed and/or direction fluctuate abruptly^2,3^. Wind shear is the term for this phenomenon. According to the International Civil Aviation Organization (ICAO), wind shear is defined as a sudden change in wind speed or direction within three nautical miles (~ 5500 m) of an airport runway threshold at an altitude of 500 m or less^4^. Changes in the aircraft's lift can be significantly impacted by wind shear. The declining and raising headwind shear on an aircraft during an approach is depicted in Fig. 2. When the approaching aircraft is subjected to a declining headwind, the lift is lowered, and the aircraft may possibly land short of the runway. In the case of a rising headwind, the aircraft's airspeed increases in relation to the surrounding air flow, generating more lift and resulting in a flatter angle of descent or even a climb, which ultimately may cause a missed approach^5^.

In addition to wind shear, turbulence may also affect an aircraft's landing. Rapid, irregular air motion is what causes turbulence. It causes jolts or hiccups. In extreme situations, the aircraft may lose control momentarily^6–8^. When an aircraft encounters turbulence, loose objects within the cabin may become dislodged, and passengers who are not wearing seat belts may sustain injuries^9^. A pilot must take corrective measures to ensure safety in the presence of significant wind shear and turbulence.

### Causes of wind shear and turbulence

**Figure 3:**
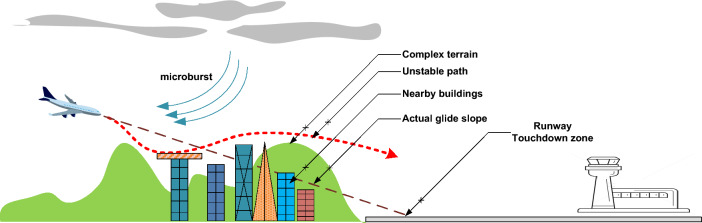
Wind Shear and Turbulence caused by nearby buildings and complex terrain.

Two basic factors causing wind shear and turbulence are meteorological elements^10,11^ and the geographical environment inside or around the airport^12–14^ as depicted in Fig. 3. Tropical cyclones, thunderstorms, cold and warm fronts, and jet streams (narrow bands of strong winds) are common weather phenomena that can cause wind shear and turbulence. Sea breezes, strong monsoon winds, and strong winds that climb over hills and man-made structures are additionally recognized to cause low-level wind shear near runways. Similarly, the intensity of turbulence is expected to decline when the wind blows over flat terrain, such as open water, and to increase when the wind blows over sizeable obstacles, such as mountains and buildings^15,16^.

### Assessment of wind shear and turbulence by numerical simulation

Over the years, various studies related to aviation meteorology have been performed to gain an understanding of low-level wind shear and turbulence. Wind shear has been assessed via computational fluid dynamics (CFD) simulations using both Reynolds Averaged Navier–Stokes (RANS) simulations and Large Eddy Simulations (LES). At Hong Kong International Airport (HKIA), Lei et al.^17^ used RANS equations and a LES model based on CFD to simulate terrain-induced wind shear. Similar to this, Chen et al.^18^ developed a high-resolution LES model using information from the Weather Research and Forecasting (WRF) model for the detection and analysis of terrain-induced low-level wind shear at HKIA. Boilley and Mahfouf^19^ performed numerical simulations with the nonhydrostatic Meso-NH model to predict the low-level wind shear over Nice airport in France. Rasheed and Sørl^20^ employed CFD analysis for the terrain-induced turbulence at Kristiansand airport, Kjevik. Zhang et al.^21^ also developed CFD-based terrain-induced wind shear models for Beijing Capital International Airport (BCIA).

Additionally, researchers have used CFD to assess turbulence intensity. Both RANS simulation and LES were used to study the transient nature of terrain-induced flow disturbances along the glide paths of descending aircraft^22^. Similar to this, Shimoyama et al.^23^ used LES to gain insight into the turbulence near Japan's Shonai Airport. According to the aforementioned studies, it has been found that the CFD model has precisely modeled wind shear and turbulence in the close vicinity of the airport. However, these studies were naturally constrained in their duration and/or scope by the exclusive use of simulation models. They employed the RANS equation, which can simulate and forecast the average airport wind field characteristics but cannot gauge the actual wind shear and turbulence intensity.

### Applications of wind tunnel studies

The wind tunnel studies offer a viable alternative to numerical simulation models for the evaluation of wind shear and turbulence characteristics surrounding airports. Consequently, the results of wind tunnel experiments can serve as a crucial basis for determining the reliability of numerical simulations. Numerous scholars from various fields have conducted wind tunnel experiments to evaluate the impact of wind on bridges, wind turbines, low- and high-rise building structures, etc. For instance, Diana et al.^24^ investigated the wind-bridge interaction by employing wind tunnel experiments and considering the aerodynamic phenomena that affect the various bridge components, primarily the deck and towers. He and Zou^25^ implemented a wind tunnel experiment to examine the aerodynamic problems that arise with train-bridge systems in the presence of wind. Li et al.^26^ studied the wind characteristics at the bridge site in the deep-cutting gorge using wind tunnel experiments. In the simulated atmospheric boundary layer, the effects of varying incoming wind directions on the wind characteristics at the bridge site were investigated.

Similarly, Su et al.^27^ demonstrated the influence of array configuration on the power performance of vertical-axis wind turbines through wind tunnel experiments. Tip speed ratio, rotational direction, and position were considered, and the performance of various array configurations was compared. Ice buildup on the blade surface of wind turbines installed in cold, humid climates significantly reduces their performance. Gao et al.^28^ conducted icing wind tunnel tests to investigate the characteristics of icing under various tip speed ratios and rime ice conditions. Likewise, using wind tunnel experiments, Cai et al.^29^ evaluated the impact of collective pitch control and cyclic pitch control on the power and loads of floating offshore wind turbines under the condition of uniform wind velocity.

Li et al.^30^ analyzed the effect of winds on the wooden pagoda in China by employing wind tunnel tests and computing the wind loads on the pagoda. One of the parts of low-rise buildings that is most susceptible to damage in windstorms is the overhang on the roof. Therefore, Wang et al.^31^ conducted field monitoring during typhoons and wind tunnel experiments to analyze the wind pressures on the upper and lower surfaces of the roof overhang in order to investigate the wind-induced pressure distributions and forces on the roof overhang of a typical low-rise building.

Despite the fact that a number of researchers have successfully employed wind tunnel experiments in their respective fields, the most serious drawback of wind tunnel investigations is the high cost of testing as well as the lack of testing infrastructure and time. To achieve the desired outcomes, numerous experiments are needed, carried out in many different kinds of settings. This reduces productivity and wastes both time and money. Empirical modeling techniques must replace experimental work in order to overcome the aforementioned limitations.

### Combining machine learning with wind tunnel studies

In recent years, engineering has made great strides with machine learning strategies^32–35^. This is a result of the growing demand for sophisticated computational methods to process enormous data sets. Several researchers have utilized it to integrate machine learning strategies with the wind tunnel outputs, such as Weng and Paal^36^ developed a novel machine learning-based wind pressure prediction model (ML-WPP) for non-isolated low-rise buildings by utilizing the non-isolated low-rise wind tunnel dataset. A machine learning-based predictive model of crosswind vibrations of rectangular cylinders was developed by Lin et al.^37^. Building pressure patterns were identified using an unsupervised machine learning method by Kim et al.^38^. Using the data collected from wind tunnel tests, deep learning methods to predict wind pressures on tall buildings were proposed^39^. Similarly, Wada et al.^40^ used deep reinforcement learning in conjunction with discrete actions to control the pitch of an aerial vehicle based on experiments in a wind tunnel.

Numerous applications have evidently led to the use of machine learning techniques to predict the wind-induced responses of structures. In contrast, its applicability to addressing the impact of wind field characteristics on airport runway glide paths is severely constrained.

### Research process

**Figure 4:**
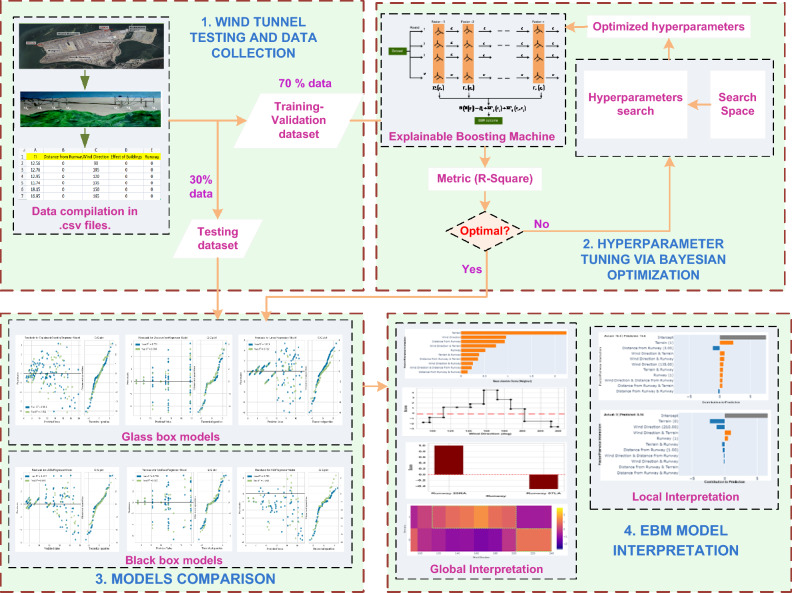
Schematic diagram of wind tunnel experiments combined with machine learning modeling.

This research combines wind tunnel experiments with machine learning models to estimate wind shear and turbulence intensity along the airport runway glide path in order to overcome the shortcomings of numerical simulations. Nevertheless, one of the most significant drawbacks of machine learning models is their black box nature. They required an explicit post-hoc explanation tool for the interpretation of the model and factors. In this study, we propose an explainable boosting machine (EBM) strategy^41^ combined with wind tunnel experiments to develop non-parametric models for estimating variation in headwind speed and turbulence intensity along the airport runway glide path. A scaled model of HKIA's north runway and Lantau Island (south of HKIA) was constructed in the TJ-3 atmospheric boundary layer (ABL) wind tunnel at Tongji University, Shanghai. Since HKIA is particularly susceptible to wind shear and turbulence due to its location and the topography of the area, an analysis of the airport's wind field is warranted^11,18^. The north runway at HKIA is oriented as 25RA and 07LA; consequently, the glide paths of 25RA and 07LA were subject to different inflow directions. Along the glide paths of both 25RA and 07LA, measurements were made at fixed distances from the runway threshold. The EBM model was trained and evaluated based on the results of wind tunnel tests to estimate variations in headwind speed and turbulence intensity. In addition, the performance of the EBM model in estimating variations in headwind speed, crosswind speed, and turbulence intensity was compared to the performance of other glass box models, including decision tree (DT)^42^ and linear regression^43^, as well as its counterpart black box models, Random Forest (RF)^44^, Extreme Gradient Boosting (XGBoost)^45^, Adaptive Boosting (AdaBoost)^46^, and Extra Tree (ET)^47^. The Bayesian optimization approach^48^ was employed during the training-validation stage to fine-tune the hyperparameters of machine learning models. Afterwards, EBM was also used to investigate a number of factors and their global and local pairwise interactions. Figure 4 depicts the entire process of wind tunnel experiments combined with machine learning modeling.

## Data preparation

### Wind field effect at HKIA

**Figure 5:**
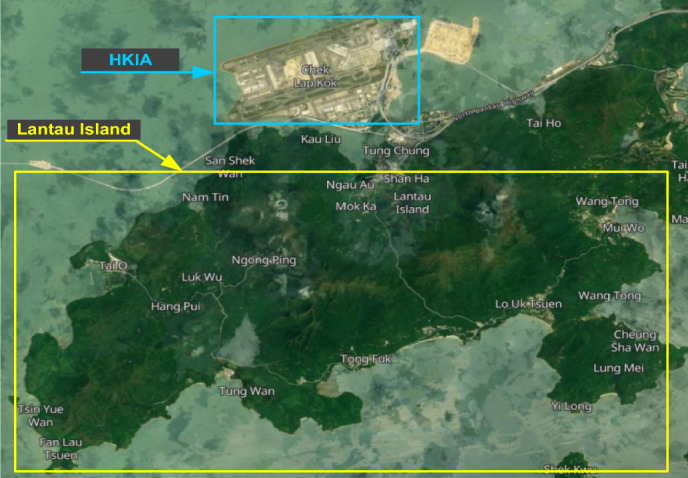
HKIA and Lantau Island.

**Figures 6:**
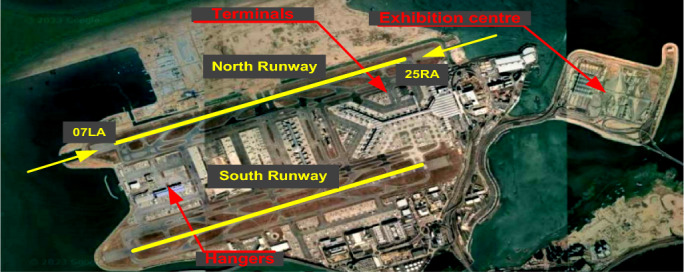
Buildings near and at HKIA.

The HKIA is located on the island of Lantau, which is subtropical terrain, off the southeast coast of the Chinese mainland as shown in Fig. 5^49^. Numerous observational and modeling studies have shown that the HKIA's complex orography and high land-sea contrast are favorable for the emergence of low-level turbulence and wind shear^50–52^. Pilot flight reports based on the HKIA indicate that nearly 1 in 500 flights have been subjected to wind shear and turbulence since the airport's opening. 97% of the pilot reports revealed 15 to 25 knots of low-level wind shear. Terrain-induced wind shear was to blame for about 70% of the wind shear that pilots reported^53^. In addition to the terrain, nearby buildings are also significant sources of wind shear and turbulence^54^, as illustrated in Fig. 6^55^.

### The wind tunnel experiments

**Figure 7:**
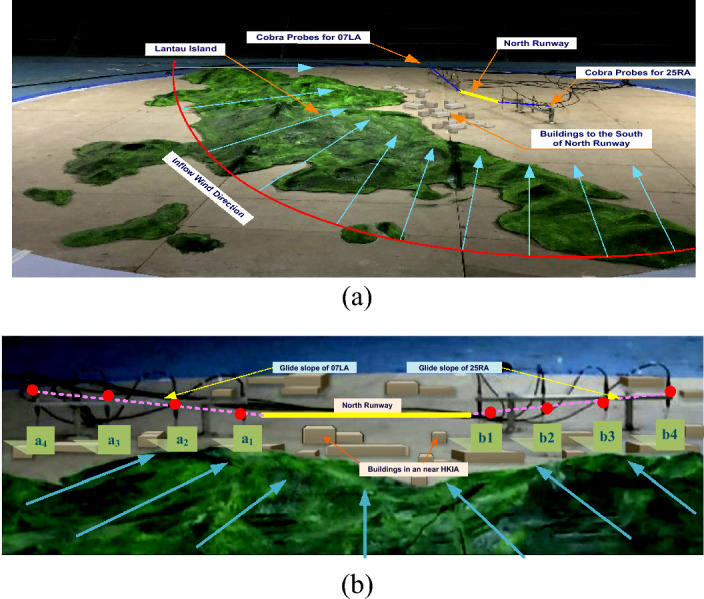
Scaled model of HKIA and Lantau Island.

This study employed wind tunnel experiments to evaluate the turbulence intensity and variations in headwind speeds along the glide path of the north runway at HKIA under various inflow wind conditions. At the State Key Laboratory for Disaster Reduction in Civil Engineering at Tongji University in Shanghai, experiments were carried out in the TJ-3 ABL wind tunnel. This wind tunnel is a low-speed, closed-circuit, vertical-return type. The wind tunnel's test chamber has dimensions of 14 m long, 15 m wide, and 2 m high. For the wind tunnel testing, Lantau Island, the north runway of the HKIA, and the buildings to the south of the runway, with a test range of 27.2 km and an average height of 425.2 m, were taken into consideration. The Lantau island and building were included in the 1:4000 scale model with a diameter of 6.8 m that was used in the wind tunnel for testing purposes, as shown in Fig. 7a. The height within the test range was 0.106 m.

The surrounding terrain model was constructed layer by layer using dense foam with a one-inch texture (equivalent to a difference in actual terrain height of 40 m). The wind tunnel blockage ratio was calculated to be 2.402%, which is less than 5% (preferably for wind tunnel research) and satisfied the requirements of the wind tunnel experiments. The wind direction was varied from 90 to 240 degrees in 15 degree increments, taking into account the typical airflow patterns of east to southeasterly winds and the southwest monsoon of Hong Kong^56^, as shown in Fig. 7b. Therefore, eleven different wind conditions were in operation. The wind directions were given as follows: 0 degrees for the north wind, 90 degrees for the east wind, 180 degrees for the south wind, and 270 degrees for the west wind. Given that aircraft usually perform headwind landings, the intervention of a southwest wind (with wind directions of 190, 210, 225, and 240 degrees) was primarily taken into account for the glide slope of Runway 25RA, and the intervention of an east-southeast wind (with wind directions of 190, 210, 225, and 240 degrees) was taken into consideration for the glide slope of Runway 07LA.

The pink dotted line in Fig. 7b shows that aircraft typically follow a glide slope of 3 degrees in the final three nautical miles before landing on the runway^57^. In wind tunnel experiments, two sets of a total of eight measurement points (a1, a2, a3, a4) and (b1, b2, b3, b4) were placed along the glide paths of runways 07LA and 25RA, respectively. The cobra probes were mounted on bespoke stands, and the installation's overall height was adjusted so that it was in line with the height of the measurement site. The probes were oriented toward the inflow direction after each test of operational conditions. Each working condition was sampled for a total of 65.536 s at a single-point sampling rate of 1000 Hz.

## Development of explainable boosting machine model

**Figure 8:**
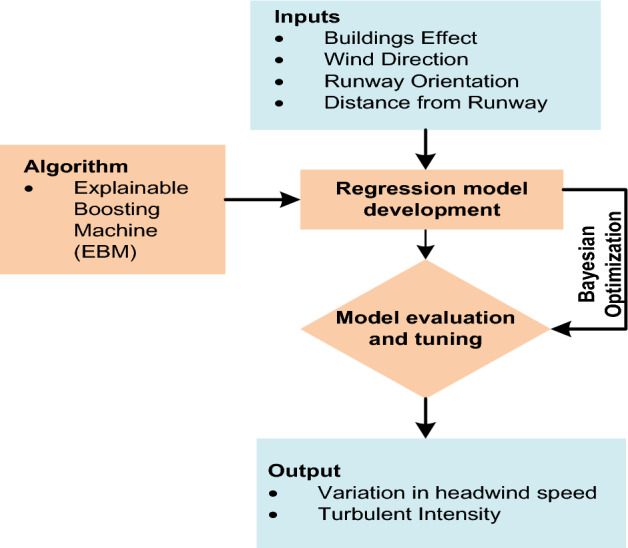
Flow chart of EBM modeling process with with input and output factors.

An explainable boosting machine (EBM) model was developed to estimate the turbulence intensity and variation in headwind along the airport runway glide slope using data from wind tunnel experiments. The EBM model is an interpretable model, in contrast to other machine learning models. When implementing machine learning models, interpretability can be crucial. By interpreting models, one can gain confidence in them and facilitate their adoption. It may also be useful for debugging the model, and in some cases, explanations for the model's predictions are required. Figure 8 depicts the EBM model, with factors serving as inputs and outputs. Prior to developing an EBM model, it is necessary to perform label encoding. The factors, which include the effect of buildings at or near HKIA, the orientation of the approach runway, and the distance from the runway, are coded as shown in Table 1.

**Table 1:** Label encoding of the factors.

Factors	Data type	Coding
Turbulence Intensity	Continuous	–
Wind shear (change in the headwind speed)	Continuous	–
Buildings effect	Discrete	1: If the effects of buildings at and near HKIA are taken into account 0: If the effects of buildings at and near HKIA are ignored
Wind direction	Continuous	–
Runway orientation	discrete	1: When using the approaching Runway 25RA's glide slope 0: When using the approaching Runway 07LA's glide slope
Distance from runway	Discrete	0: When the distance between the aircraft and the end of the approaching runway is 0.25 nautical miles (0.25MF) 1: When the distance between the aircraft and the end of the approaching runway is 0.75 nautical miles (0.75MF) 2: When the distance between the aircraft and the end of the approaching runway is 1.25 nautical miles (1.25MF) 3: When the distance between the aircraft and the end of the approaching runway is 1.75 nautical miles (1.75MF)

### Fundamental concept of EBM model

**Figure 9:**
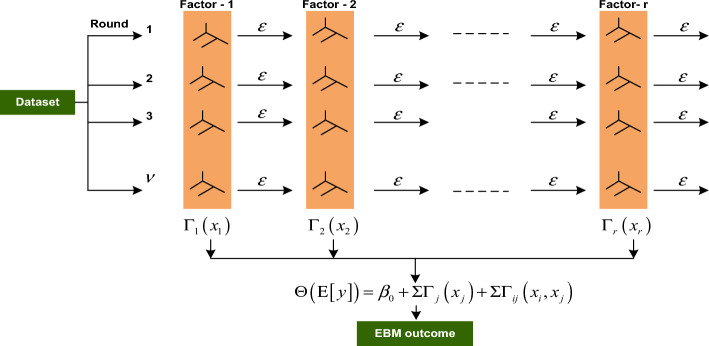
Working principle of EBM algorithm.

The EBM algorithm is based on GAMs, which are universally acknowledged to be the gold standard for comprehensibility. Given that $\Delta = \left( {x_{j} ,y_{j} } \right)$ is a training dataset with a length of $R$, that $\left( {x_{1} ,x_{2} ,...,x_{R} } \right)$ are the input vectors with $v$ number of factors, and that $y_{r}$ is the target factor, then the GAMs takes the form as shown in Eq. (1).

1 $$ \Theta \left( {E\left[ y \right]} \right){ = }\beta_{{\text{o}}} { + }\sum {\Gamma_{j} } \left( {x_{j} } \right) $$

where, $x_{j}$ indicates the *j*th factor in the factors set, $\Theta$ is the link function that conforms the GAM to regression (e.g., $\Theta$ = identity) or classification (e.g., $\Theta$ = logistic), and $\Gamma_{r}$ is the attribute function. EBM, which employs bagging and gradient boosting, provides several significant advantages over conventional GAMs. An adequate description would be bagged, augmented, and lowered shallow trees. As depicted in the Fig. 9, the core of the algorithm employs boosted trees.

In EBM, training is carried out repeatedly, with each repetition involving the creation of a distinct boosting procedure for each factor. The factor's order is irrelevant because of the low learning rates used in this process. In order to determine the best way to determine the contribution of each factor to the model's prediction, the high number of iterations aims to reduce the effects of co-linearity. Additionally, pairwise interaction terms can be automatically detected and included by EBM, improving the model's accuracy while maintaining its explainability. The fact that EBM is an additive model allows for the capture and visualization of each factor's contribution, which improves explainability.

Small trees are built sequentially for each iteration of the model, each of which can only use one factor. In a boosting manner, the residual $\left( \varepsilon \right)$ is updated and a new tree is built using a different factor. Every iteration involves doing this for every factor. When training is finished, we can view all the trees that a particular factor used to build them. Due to the concepts of additivity and modularity, it is feasible to order and visualize the contributions to determine which factor had the greatest influence on the individual prediction. Another advantage of the EBM is the ability to improve accuracy by incorporating pairwise interactions into standard GAMs that becomes GA2Ms with the form and given as Eq. (2).

2 $$ \Theta \left( {E\left[ y \right]} \right){ = }\beta_{{\text{o}}} { + }\sum {\Gamma_{r} } \left( {x_{r} } \right) + \sum {\Gamma_{rs} } \left( {x_{r} ,x_{s} } \right) $$

Here, the two-dimensional interaction $\Gamma_{rs} \left( {x_{r} ,x_{s} } \right)$ can be depicted as a heat map on the two-dimensional $x_{r} - x_{s}$ plane while still retaining an exceptionally high degree of intelligibility. Initially, GA2M constructs the best GAM possible, and then it searches through the residuals to find all of the possible combinations of interactions, and then it ranks them based on how crucial they are. The last step is to integrate into the model the $\kappa$ most significant pairwise interactions, where $\kappa$ is a value determined through the process of cross-validation.

### Hyperparameter tuning of EBM via Bayesian optimization

In this study, the process of fine-tuning the hyperparameters of EBM model as well as other counterpart black box and other glass box model was carried out with the aid of a Bayesian optimization strategy. It is one of the algorithms for global optimization, and it finds widespread application in the field of engineering^58,59^. The objective function that needs to be optimized in order to find the optimal values for the hyperparameters of the model can be expressed as Eq. (3).

3 $$ x^{\Theta } = \mathop {\arg \max }\limits_{{x \in \Lambda_{{\text{s}}} }} \varphi \left( x \right) $$

where, $x$: Hyperparameters of the model, $\Lambda_{s}$: Hyperparameters search space, $\varphi \left( x \right)$: Objective function that reflects the association between models’ performance and hyperparameters.

In this study, we employed *R*^*2*^ as the evaluation metric. The purpose of this optimization is to identify the appropriate combination of hyperparameters $\left( {x^{\Theta } } \right)$ in such a way that the performance of the model $\varphi \left( x \right)$ is increased to its full potential. The concepts of the Bayesian theorem are utilized in Bayesian optimization, which includes the following [Eq. (4)].

4 $$ \rho \left( {\varphi \left| {\rm T} \right.} \right) = \frac{{\rho \left( {{\rm T}\left| \varphi \right.} \right)\rho \left( \varphi \right)}}{{\rho \left( {\rm T} \right)}} $$

where, $\varphi$: Black box function, $\rho \left( \varphi \right)$: Prior probability of $\varphi$, $\rho \left( {{\rm T}\left| \varphi \right.} \right)$: Probability obtained from the observed point, $\rho \left( {\rm T} \right)$: Normalized constant, $\rho \left( {\varphi \left| {\rm T} \right.} \right)$: Given the current observation point ${\rm T}$, the posterior probability of $\varphi$.

If the Gaussian process is used, then the black box function $\varphi$ will behave in a way that is consistent with the Gaussian distribution. At this very moment, the acquisition function $\Upsilon \left( x \right)$, which is based on the expected improvement, looks like this:

5 $$ \Upsilon \left( x \right) = \left\{ {\begin{array}{*{20}l} {\left( {\upsilon \left( x \right) - \varphi^{ + } } \right)\Lambda \left( Z \right) + \alpha \left( x \right)\beta \left( Z \right)} \hfill & {\alpha \left( x \right){ > 0}} \hfill \\ {\max \left( {0, \, \upsilon \left( x \right) - \varphi^{ + } } \right)} \hfill & {\alpha \left( x \right) = 0} \hfill \\ \end{array} } \right. $$

6 $$ Z = \frac{{\upsilon \left( x \right) - \varphi^{ + } }}{\alpha \left( x \right)} $$

where, $\Lambda$: Cumulative distribution function of Gaussian distribution, $\beta$: PDF of Gaussian distribution

The process of Bayesian optimization of hyperparameters, the iterative search is as follow:

Iterate the loop $\omega$ times $(\omega = 1,2, \ldots ,):$

1. In accordance to the acquisition function $\Upsilon \left( x \right)$ computed by Eq. (4), the hyperparameters combination $x_{\omega + 1} = \arg \max \Upsilon \left( {x;{\rm T}} \right)$ of the next cluster of instances is obtained.
2. Corresponds to an instance $x_{\omega + 1}$, compute the performance $\varphi_{\omega + 1}$ of the machine learning model.
3. Update $x_{\omega + 1}$ to the observed point ${\rm T}_{1:\omega + 1} = \left\{ {{\rm T}_{1:\omega } ,\left( {x_{\omega + 1} ,\varphi_{\omega + 1} } \right)} \right\};$
4. The posterior distribution of $\varphi$ is then updated in accordance to $\rho \left( {\varphi \left| {\rm T} \right.} \right) = \frac{{\rho \left( {{\rm T}\left| \varphi \right.} \right)\rho \left( \varphi \right)}}{{\rho \left( {\rm T} \right)}}$

When the loop terminates, the final obtained combination of hyperparameters is the output of Bayesian optimization.

### Performance measures

To assess the performance of different models, four distinct metrics could be utilized, i.e., mean absolute error (MAE), mean squared error (MSE), root mean squared error (RMSE), and Coefficient of determination (*R*^2^). The MAE is the average absolute value of the individual prediction errors across all instances [Eq. (7)]. MSE is calculated using the average squared difference between observed and predicted values, as shown in Eq. (8). RMSE is the square root of the difference between the observed and predicted values, according to Eq. (9). R^2^ which ranges from 0 to 1, indicates the ability of a model to accurately predict values. R^2^ is given by Eq. (10).

7 $$ MAE = \sum\nolimits_{x = 1}^{\zeta } {\frac{{\left| {\sigma_{x} - \overline{\sigma }_{x} } \right|}}{\zeta }} $$

8 $$ MSE = \frac{1}{\zeta }\sum\nolimits_{x = 1}^{\zeta } {\left( {\sigma_{x} - \overline{\sigma }_{x} } \right)}^{2} $$

9 $$ RMSE = \sqrt {\sum\nolimits_{x = 1}^{\zeta } {\frac{{\left( {\sigma_{x} - \overline{\sigma }_{x} } \right)^{2} }}{\zeta }} } $$

10 $$ R^{2} = 1 - \frac{{\sum\nolimits_{x = 1}^{\zeta } {\left( {\sigma_{x} - \overline{\sigma }_{x} } \right)^{2} } }}{{\sum\nolimits_{x = 1}^{\zeta } {\left( {\sigma_{x} - \sigma_{avg} } \right)^{2} } }} $$

where, the total number of data points are denoted by ζ, the actual experimental outcomes are represented by *σ*_*x*_, the predicted outcomes are represented by $\overline{\sigma }_{x}$ and *σ*_*avg*_ represents the average of predicted outcomes.

## Results and discussion

**Figure 10:**
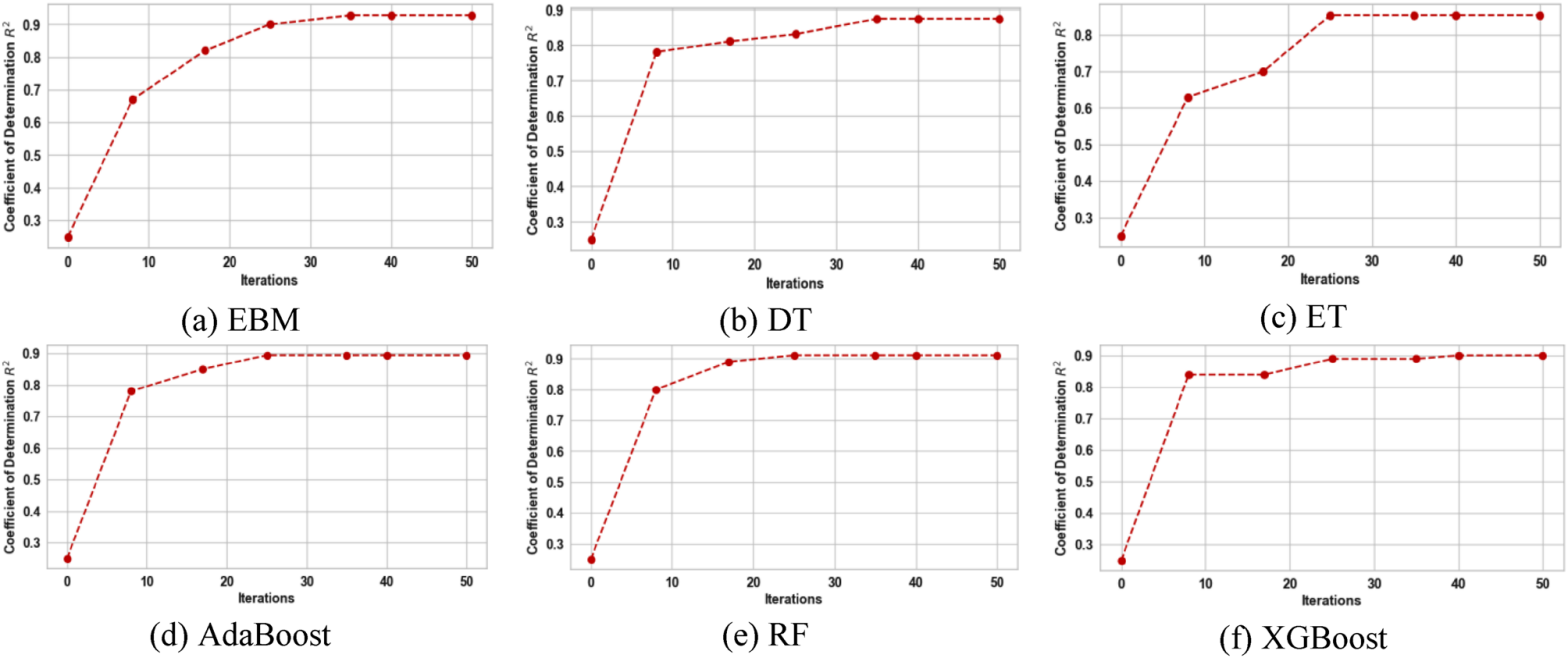
Progress of Bayesian optimization process for variation in headwind speed estimation.

**Figure 11:**
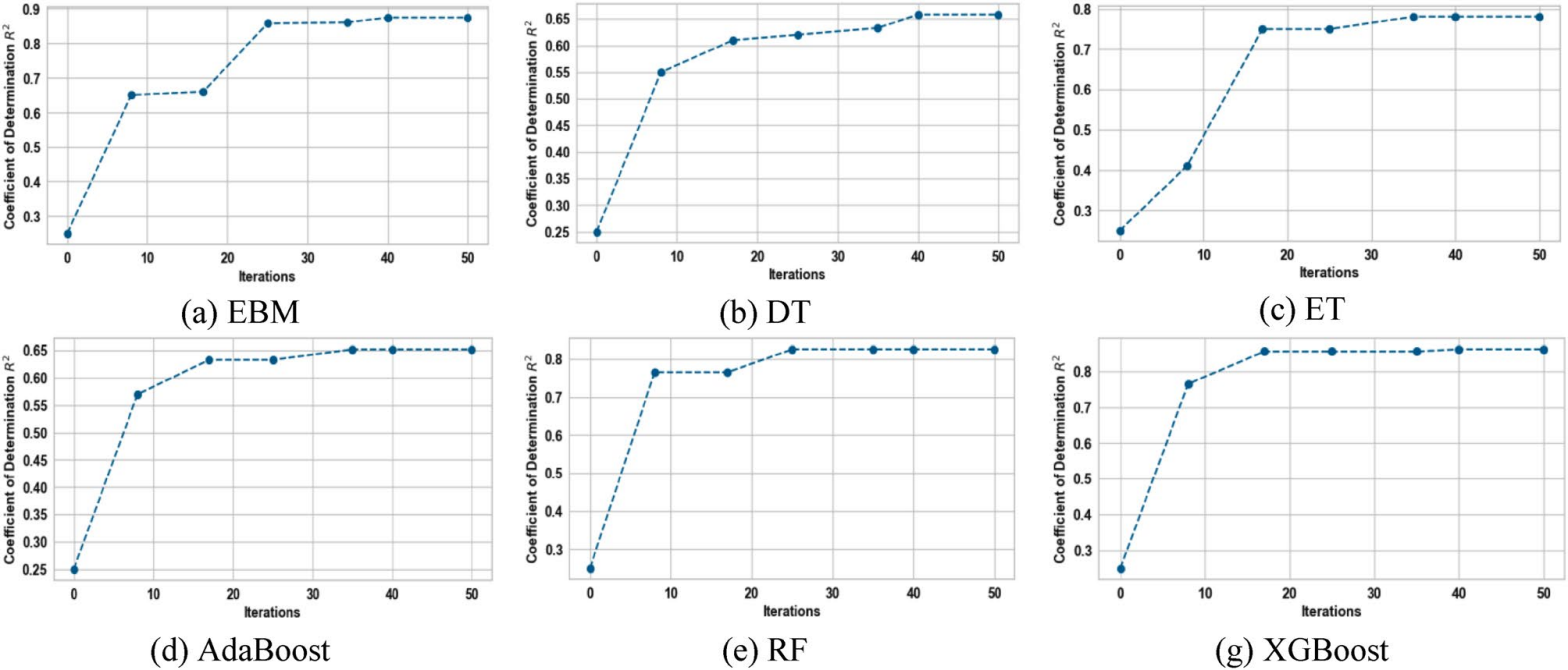
Progress of Bayesian optimization process for the estimation of turbulence intensity.

This section presents the development of the EBM model, other glass box models, such as DT and linear regression, and their counterpart black box models, such as RF, XGBoost, AdaBoost, and ET, to estimate variation in headwind speed and turbulence intensity based on data from wind tunnel experiments. In order to achieve better performance, it is crucial to note that the hyperparameters must be optimized prior to the development of these models. To achieve this, the results of the wind tunnel experiments were split into a training-validation set (comprising 70% of the total) and a testing set (comprising 30% of the total)^60^. The development of machine learning models and the tuning of their hyperparameters through Bayesian optimization were both done using the training-validation data. Bayesian optimization aimed to find the set of hyperparameters for various machine learning models that maximizes the R^2^ value across a given sample space. Figures 10 and 11 show the progress of the Bayesian optimization process over 50 iterations using both glass box and black box models for variations in headwind speed and turbulence intensity, respectively. They were tuned using the hyperparameters associated with the models at the best R^2^ to provide the best prediction model. Table 2 shows the optimal values for the hyperparameters that were obtained for the estimation of variation in headwind speeds, while Table 3 shows the optimal hyperparameters for models that estimate turbulence intensity.

**Table 2:** Optimal hyperparameters for the estimation of variation in the headwind speeds.

Model	Algorithm	Hyperparameters	Range	Optimal values
Glass box	EBM	learning_rate	(0.01, 0.5)	0.35
		min_sample_leaf	(3, 10)	5
	DT	max_depth	(2, 10)	3
Black box	ET	max_depth	(2, 10)	4
		n_estimators	(10, 100)	50
	RF	max_depth	(2, 10)	3
		n_estimators	(10, 100)	62
	XGBoost	n_estimators	(10, 100)	38
		learning_rate	(0.01, 0.5)	0.18
	AdaBoost	n_estimators	(10, 100)	25
		learning_rate	(0.01, 0.5)	0.19

**Table 3:** Optimal hyperparameters for the estimation of turbulence intensity.

Model	Algorithm	Hyperparameters	Range	Optimal values
Glass box	EBM	learning_rate	(0.01, 0.5)	0.42
		min_sample_leaf	(3, 10)	4
	DT	max_depth	(2, 10)	3
Black box	ET	max_depth	(2, 10)	3
		n_estimators	(10, 100)	25
	RF	max_depth	(2, 10)	4
		n_estimators	(10, 100)	20
	XGBoost	n_estimators	(10, 100)	17
		learning_rate	(0.01, 0.5)	0.19
	AdaBoost	n_estimators	(10, 100)	15
		learning_rate	(0.01, 0.5)	0.14

### Prediction of variation in headwind speed and turbulence intensity

Once the optimal hyperparameters were determined, holdout or testing data was used to evaluate and compare the performance of various models. Importantly, the dataset was partitioned after being shuffled with 40% and 50% test data, and this demonstrated that the metrics values for model testing remained constant within the 95% confidence interval. In addition to tuning hyperparameters, this was performed to prevent over-fitting. When the size of the test data was altered, no anomalies in the performance metrics were observed. Table 4 presents the performance metrics of various models for estimating the variation in headwind speeds using both the training and test datasets. In terms of MAE (0.346), MSE (0.189), RMSE (0.435), and R2 (0.928) for the training dataset and MAE (0.571), MSE (0.466), RMSE (0.682), and R2 (0.839) for the testing dataset, the EBM model performed better than other glass box models. Similarly, the XGBoost model outperformed other black box models using testing data, with an MAE of 0.830, an MSE of 0.926, an RMSE of 0.964, and an R2 of 0.680. Overall, the EBM model performed better than its black-box counterpart. The relative percent difference between MAE of EBM and its counterpart XGBoost on the testing dataset was 36.974%, MSE was 66.092%, RMSE was 32.265%, and R2 was 21.053%.

**Table 4:** Performance measures of different models for estimating variation in headwind speeds.

Transparency	Models	Training dataset				Testing dataset			
		MAE	MSE	RMSE	*R*^*2*^	MAE	MSE	RMSE	*R*^2^
Glass box	EBM	0.346	0.189	0.435	0.928	0.571	0.466	0.682	0.839
	LR	2.861	10.965	3.316	0.268	2.475	9.716	3.116	0.370
	DT	0.424	0.332	0.579	0.874	0.925	1.194	1.091	0.589
Black box	ET	0.495	0.390	0.625	0.852	0.858	0.984	0.994	0.659
	AdaBoost	0.388	0.284	0.0535	0.894	0.931	1.210	1.105	0.584
	RF	0.336	0.152	0.396	0.911	0.828	1.074	1.037	0.630
	XGBoost	0.353	0.250	0.504	0.905	0.830	0.926	0.964	0.680

Table 5 enumerates the outcomes of various models' attempts to estimate the turbulence intensity for the training and test datasets. The EBM model performed better compared to both the black box and the glass box models, with an MAE of 1.705, MSE of 3.994, RMSE of 1.993, and *R*^2^ of 0.741, using the testing dateset. The LR model performed the worst overall, with MAE, MSE, RMSE, and *R*^2^ values of 2.516, 9.664, 3.109, and 0.209, respectively.

**Table 5:** Performance measures of different models for the estimation of turbulence intensity.

Transparency	Models	Training dataset				Testing dataset			
		MAE	MSE	RMSE	*R*^*2*^	MAE	MSE	RMSE	*R*^2^
Glass box	EBM	1.051	1.887	1.373	0.874	1.705	3.991	1.993	0.741
	LR	2.901	11.056	3.328	0.317	2.516	9.668	3.109	0.206
	DT	1.807	5.533	2.352	0.658	2.237	7.184	2.685	0.410
Black box	ET	1.442	3.253	1.806	0.781	1.755	4.665	2.158	0.695
	AdaBoost	1.925	5.626	2.372	0.652	1.726	4.694	2.166	0.614
	RF	1.178	2.615	1.6171	0.825	1.609	4.579	2.132	0.706
	XGBoost	1.115	2.246	1.497	0.861	1.490	3.586	1.894	0.705

**Figure 12:**
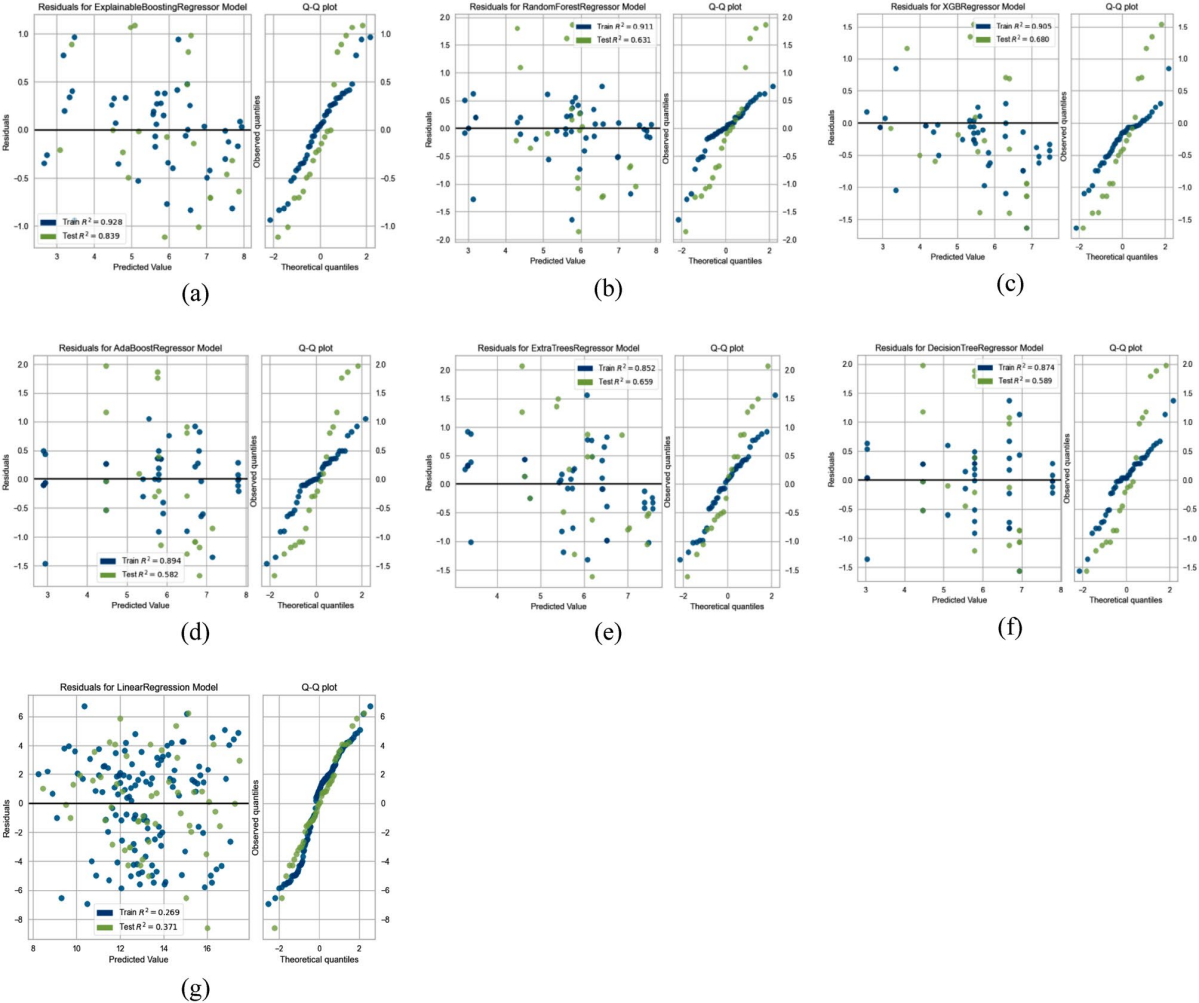
Residual analysis in case of variation in headwind speeds; Glass box models (**a**–**c**), Black box models (**d**–**g**).

**Figure 13:**
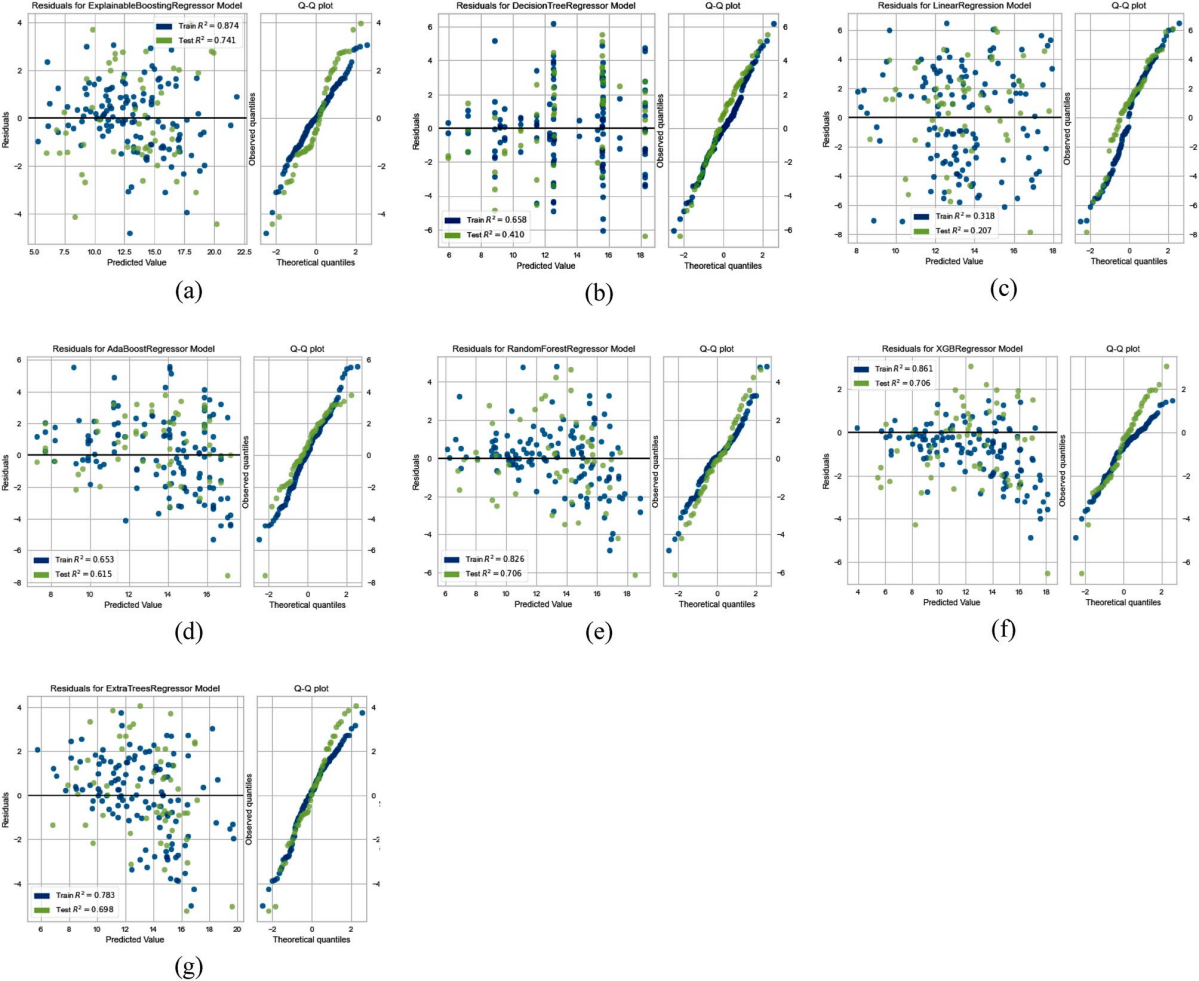
Residual analysis in case of turbulence intensity; Glass box models (**a**–**c**), Black box models (**d**–**g**).

Similarly, in the case of both variations in headwind speed and turbulence intensity, we also reported and compared the prediction confidence and their ability to extrapolate on unseen data for both black box and glass box models using a residual scatter plot and a residual QQ (quantile–quantile) plot, as shown in Figs. 12 and 13. By analyzing the residual scatter plot's residual patterns, the model's applicability is shown. The discrepancy between the predicted and observed values is represented by residues. In a residual scatter plot, the target factor values are plotted on the horizontal axis, and the residuals are plotted on the vertical axis. When using the EBM model to analyze residuals for variations in headwind speed as well as turbulence intensity, in contrast to other models, the points are closer to the horizontal line at 0.00.

### EBM interpretation

Based on its performance metrics utilizing a testing dataset, EBM was inevitably the best model. From the EBM model, we can glean explanations. Consequently, we next utilized the EBM model to illustrate global and local interpretations of various individual and pairwise factor interactions.

#### Global importance of factors and pairwise interaction

The EBM algorithm is a generalized additivity model that enhances and expands the tree-based model. Due to the persistence of additivity, the contributions of factors can be ranked and plotted to demonstrate impact prediction in both global and local contexts. The exhaustive global explanation of the EBM makes it possible to visualize the effect of each possible combination of factors on the variation in headwind speed and turbulence intensity.

**Figure 14:**
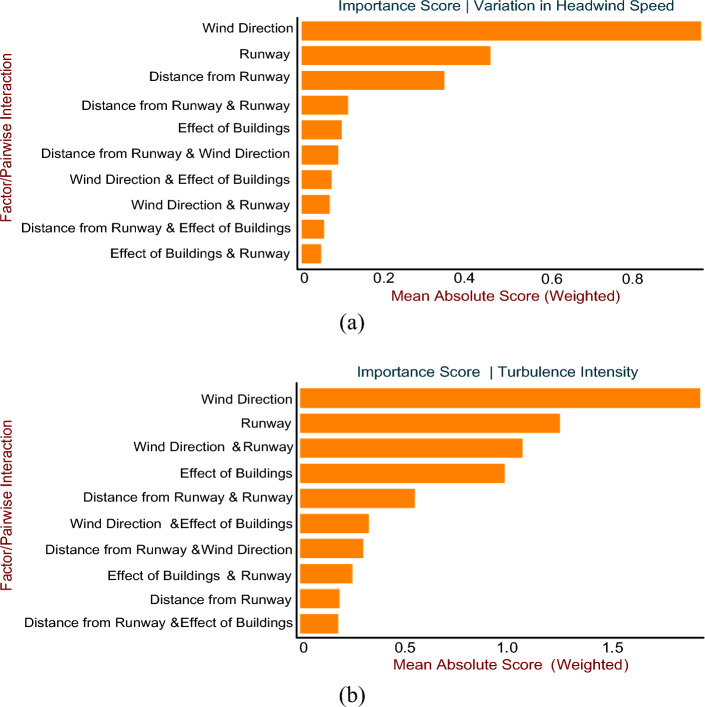
Global Importance of factors and their pairwise interaction; (**a**) In case of variation in headwind speed; (**b**) In case of turbulence intensity.

Figure 14a and b provides a summary of the global significance of each individual factor and their pairwise interactions for variation in headwind speed and turbulence intensity, respectively. Wind direction contributed the most to the variation in headwind speed, followed by Runway. In pairwise interaction, the most significant contributor to the variation in headwind speed was the combination of Distance from Runway and Runway. Similarly, wind direction contributed the most to turbulence intensity, followed by runway length and distance from the runway. The combination of wind direction and runway contributed the most to the occurrence of intense turbulence in pairwise interaction. These results are also inline with the previous study^54,61^

**Figure 15:**
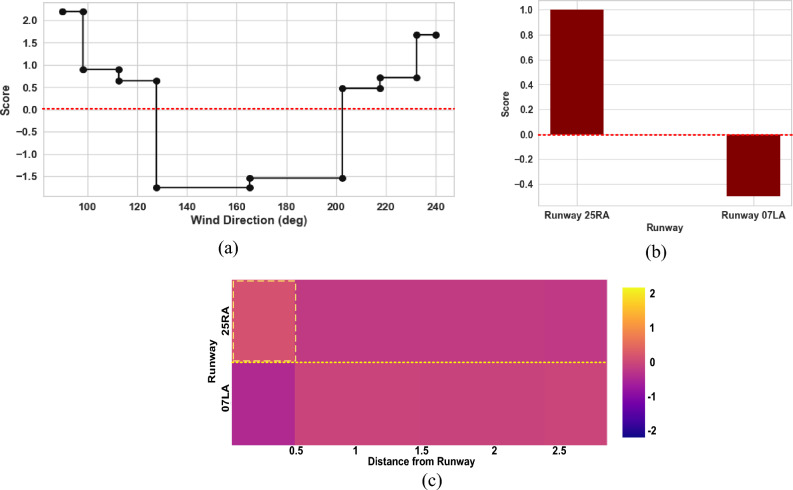
Shape function and heat map obtained via EBM model for mean wind speed; (**a**) shape function for Distance from Runway factor; (**b**) shape function for wind direction; (**c**) heat map for pairwise interaction of Runway and Distance from Runway factors.

**Figure 16:**
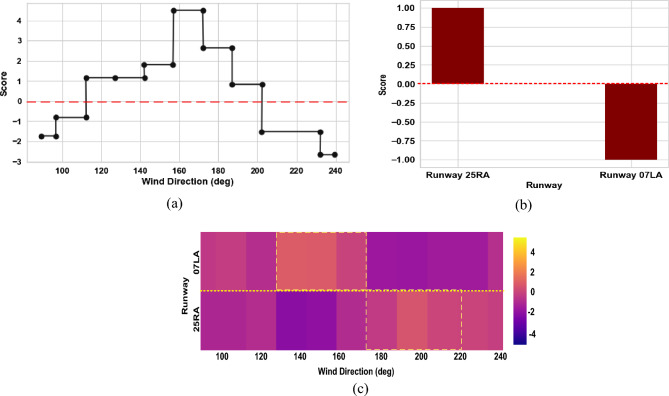
Shape function and heat map obtained via EBM model for turbulence intensity; (**a**) shape function for wind direction factor; (**b**) shape function for Runway; (**c**) heat map for pairwise interaction of Runway and wind direction factors.

Additional output provided by the proposed EBM model for comprehending the global findings for the variation in headwind speed and turbulence intensity is shown as examples in Figs. 15 and 16, respectively. These include EBM shape functions and a heat map. For each predictor factor, the gradient boosting procedure yields a large number of decision trees, which are then used to generate the function characterizing the dependent factor's response to the specific predictor factor. One can then graphically represent these functions as a one-dimensional function, with the values of the predictor factors along the horizontal axis and the scores (i.e., the effect of the predictor factor on the prediction) along the vertical axis. When the score is greater than 0, it means that the value of the predictor factor is strongly linked to the outcome. In the case of the variation in headwind speed, Fig. 14a–c shows EBM shape functions and a heat map for the important individual and pairwise interaction factors.

Figure 15a demonstrates that the variation in headwind speed is greatest when the wind direction is between 90° and 135°, as well as between 200° and 240°. The headwind speed along the glide path of Runway 25RA significantly decreases due to the presence of buildings at the airport that are in its path. Runway 25RA persists in being affected by a wind coming from the southwest at an angle ranging from 195° to 240°. Compared to Runway 07LA, Runway 25RA is more likely to experience a greater variation in headwind speed (Fig. 15b). This may be due to the presence of buildings close to Runway 25RA that cause variations in wind speed.

The pairwise interactions between runway and distance from runway are depicted in Fig. 15c. The heat map revealed that the variation in headwind speed is greatest within 0.5 miles of Runway 25RA. Consequently, shorter distances along Runway 25RA have been more susceptible to variations in headwind speed, and pilots may encounter severe wind shear in this area. Pilots must be extremely vigilant during the final approach to Runway 25RA.

Figure 16a–c displays EBM shape functions and a heat map for the crucial individual and pairwise interaction factors in the case of the turbulence intensity. The higher turbulence intensity is more likely to occur for wind directions between 110° and 200°, as shown in Fig. 16a. Complex terrain in this direction blocks the wind, causing it to fluctuate in speed, which ultimately increases the intensity of the turbulence. In contrast to Runway 07LA, Runway 25RA is more likely to encounter higher turbulence intensity than Runway 07LA (Fig. 16b). This may be caused by the presence of building structures close to Runway 25RA that cause wind speed variations. This suggests that nearby structures are restricting the wind close to the runway^62^. The heat map in Fig. 16c shows how the runway and wind direction interact pairwise. It demonstrates that Runway 25RA is most likely to experience high turbulence intensity when the wind direction is between 170 and 220 degrees and that Runway 07LA is most likely to experience high turbulence intensity when the wind direction is between 130 and 170 degrees.

#### Local factor interpretation

The local explanation, which includes the explanations behind particular predictions in a single case, can be investigated for both variations in headwind speeds and turbulence intensity. The intercept is shown to be constant in gray, while additive terms that have a positive effect on output are shown in orange, and additive terms that have a negative effect on output are shown in blue, demonstrating local factor contribution assessments for each instance. The estimates for each instance are derived from the shape functions and pairwise interactions (heat map) based on the input values. We take into account two arbitrary cases where the predictions are very close to actual values, each for variations in headwind speed and turbulence intensity. Nevertheless, we can perform local interpretation in other instances as well.

**Figure 17:**
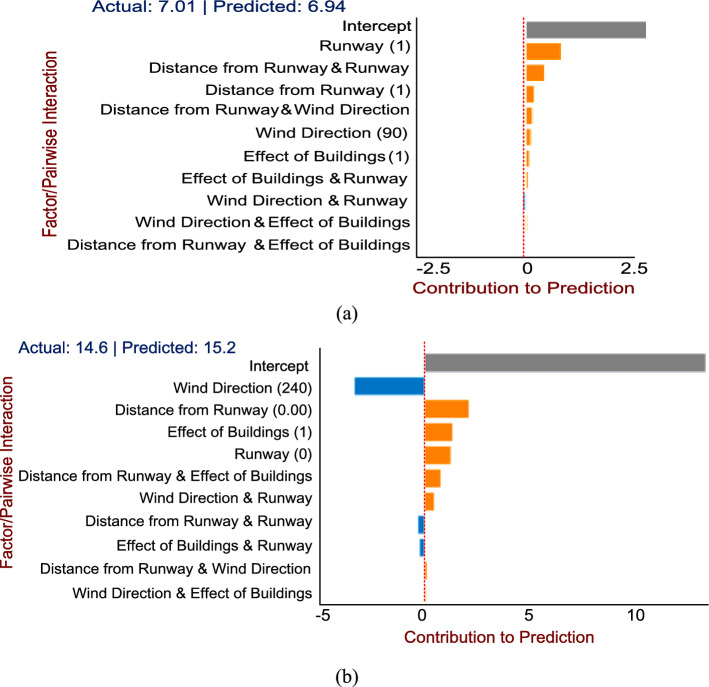
Local Interpretation for randomly selected instances; (**a**) In case of variation in headwind speed; (**b**) In case of turbulence intensity.

The local explanation for a variation in headwind speed prediction is shown in Fig. 17a. It demonstrates that the variation in mean headwind speed was high for a randomly chosen instance at Runway 25RA and a shorter distance from the runway threshold. Similarly, Fig. 17b shows the degree of turbulence for a randomly selected instance, showing that a wind direction of 240 degrees has a negative effect on the turbulence intensity. However, a closer proximity to the runway, the presence of buildings, and Runway 25RA all increase the likelihood of experiencing high turbulence.

## Conclusion and future work

In order to estimate the variation in headwind speed and turbulence intensity along the airport runway glide slope based on wind tunnel experiments, this study employs an explainable boosting machine (EBM), a state-of-the-art glass box model. The proposed approach is both explicable and intuitive, and it provides accuracy on par with black box competitors. Conventional machine learning regression models lack explainability, but the EBM model has been effective in overcoming this limitation. Therefore, the following conclusions can be drawn:

- Based on the testing data set for the variation in headwind speed, the Bayesian optimized EBM model had the best overall performance of all the black box and glass box models, with an MAE of 0.571, an MSE of 0.466, an RMSE of 0.682, and an R^2^ of 0.839. Similarly, in the case of holdout dataset turbulence intensity, the Bayesian optimized EBM model outperformed other models with an MAE (1.705), MSE (3.991), RMSE (1.993), and R^2^ (0.741).
- The LR model demonstrated the worst performance with MAE (2.475), MSE (9.716), RMSE (3.116) and *R*^2^ (0.370) in case of variation in headwind speed estimation as well as in estimating turbulence intensity with MAE (2.516), MSE (9.668), RMSE (3.109) and *R*^2^ (0.206).
- In the case of individual factors for the variation in headwind speed, EBM-based global interpretation revealed that “wind direction” and “Runway” significantly contributed to the occurrence of high variation in headwind speed. In the case of pairwise interaction, it was found that the combination of "distance from runway" and "runway" contributed more to the high variation in headwind speed.
- The EBM-based interpretation also demonstrated that, in the case of individual factors, "Wind Direction" and "Runway" significantly influenced the occurrence of high turbulence intensity. It was found that the combination of "Wind Direction" and "Runway" contributed more to the high turbulence intensity in the case of pairwise interaction.

This proposed EBM framework could be applied to a comprehensive analysis of wind field characteristics at other airports as well. It is indeed a great resource for individuals associated with civil aviation. However, there are a few recommendations for future research.

- Although this study utilized a number of different input parameters to predict wind field characteristics along the airport runway glide slope, many other parameters, such as atmospheric pressure and temperature, could be considered in future studies.
- In this study, the variation in headwind speed and turbulence intensity along the airport runway glide slopes were the parameters of concern. Similarly, the turbulence integral length scale as well as the variation in crosswind speeds at different points on the glide path can also be considered for future work.

## Data Availability

The datasets used and analyzed in the current study is available from the corresponding author on reasonable request.
